# Day-Time Declamping Is Associated with Better Outcomes in Kidney Transplantation: The Circarein Study

**DOI:** 10.3390/jcm10112322

**Published:** 2021-05-26

**Authors:** David Montaigne, Nasser Alhawajri, Mathilde Jacquelinet, Amandine Coppin, Marie Frimat, Sébastien Bouyé, Gilles Lebuffe, Bart Staels, Christian Jacquelinet, Marc Hazzan

**Affiliations:** 1 Univ. Lille, Inserm, CHU Lille, Institut Pasteur de Lille, U1011–EGID, 59000 Lille, France; mathilde.jacquelinet@chru-lille.fr (M.J.); amandine.coppin@chru-lille.fr (A.C.); bart.staels@pasteur-lille.fr (B.S.); 2 Renal Epidemiology and Information Network (REIN) Biomedecine Agency, 93212 Saint-Denis, France; nasser.alhawajri@biomedecine.fr (N.A.); christian.jacquelinet@biomedecine.fr (C.J.); 3 CHU Lille, 59000 Lille, France; marie.frimat@chru-lille.fr (M.F.); sebastien.bouye@chru-lille.fr (S.B.); gilles.lebuffe@chru-lille.fr (G.L.); marc.hazzan@chru-lille.fr (M.H.); 4 Inserm U1018, 94807 Villejuif, France

**Keywords:** kidney transplantation, transplant survival, ischemia-reperfusion, time-of-the day

## Abstract

Despite improvements in organ preservation techniques and efforts to minimize the duration of cold ischemia, ischemia–reperfusion (IR) injury remains associated with poor graft function and long-term survival in kidney transplantation. We recently demonstrated a clinically significant day-time variation in myocardial tolerance to IR, transcriptionally orchestrated by the circadian clock. Patient and graft post-transplant survival were studied in a cohort of 10,291 patients first transplanted between 2006 and 2017 to test whether kidney graft tolerance to IR depends on the time-of-the-day of clamping/declamping, and thus impacts graft and patient survival. Post-transplant 1- and 3-year survival decreased with increasing ischemia duration. Time-of-the-day of clamping did not influence outcomes. However, night-time (vs. day-time) declamping was associated with a significantly worse post-transplant survival. After adjustment for other predictors, night-time (vs. day-time) declamping remained associated with a worse 1-year (HR = 1.26 (1.08–1.47), *p* = 0.0028 by Cox multivariable analysis) and 3-year (HR = 1.14 (1.02–1.27), *p* = 0.021) outcome. Interestingly, the deleterious impact of prolonged ischemia time (>15 h) was partially compensated by day-time (vs. night-time) declamping. Compared to night-time declamping, day-time declamping was associated with a better prognosis of kidney transplantation despite a longer duration of cold ischemia.

## 1. Introduction

Despite improvements in organ preservation techniques and efforts to minimize the duration of cold ischemia, ischemia-reperfusion (IR) injury remains associated with poor graft function and long-term survival in kidney transplantation [1]. Kidney transplantation is hence performed irrespective of the time of the day to keep the cold ischemia period as short as possible. However, off-hour surgery may result in lower performance of medical care due to staff fatigue. The net result of these two opposing factors on outcomes has only been explored in a few small studies focusing on graft declamping time-of-the-day, which reported inconsistent, and sometimes counter-intuitive results (e.g., less technical failure at night) [2,3,4,5,6,7,8]. Importantly, time-of-the-day has been demonstrated to impact organ tolerance to IR injury [9]. Our group recently demonstrated a clinically significant day-time variation in myocardial tolerance to the controlled IR insult imposed during cardiac surgery, with patients undergoing aortic valve replacement in the afternoon displaying lower peri-operative myocardial injury and post-operative morbidity than those operated on in the morning [10], through mechanisms implying the myocardial circadian clock. In line, studies using clock gene-deficient mice revealed a role of the renal clock machinery in kidney IR tolerance, with circadian clock gene and nuclear receptor RORα signaling being protective against renal IR injury [11]. Whether such a circadian clock-driven variation in kidney IR tolerance exists in the transplanted graft and impacts on transplantation outcome is unknown.

Therefore, whether there is an optimal time-of-the-day for kidney transplantation remains to be determined, i.e., whether clamping and/or declamping times are associated with post-transplant outcomes, independently of ischemia duration.

We thus retrospectively studied, using the database of the French National Registry of Organ Transplantation (Cristal, Agence de la Biomédecine), the impact of time-of-the-day of clamping and declamping, as well as their interaction with clamping duration, on mortality and graft survival after deceased donor renal transplantation.

## 2. Methods

### 2.1. Study Design

A retrospective cohort analysis was performed after approval in 2017 to evaluate the impact of clamping and declamping time on the composite endpoint “patient and graft survival” 1 and 3 years after renal transplantation. Moreover, using a paired analysis study design, post-transplantation outcomes were also analyzed for the two kidney allografts donated from the same deceased donor and transplanted in two different recipients. This method eliminates the effects of donor factors on transplantation outcomes.

### 2.2. Population

Patients were selected from the French Registry of Organ Transplantation (Cristal registry), which registers data on all donor and transplant recipients in France [12].

All first kidney transplant recipients from brain dead donors between January 2006 and December 2017 were included. Exclusion criteria were: Pediatric recipients (<18 years-old), multiple organ transplanted patients, living-donor recipients, non-heart-beating donor recipients, and re-transplanted patients.

From this initial cohort, only patients with reliable cross-validated ischemia duration, clamping and declamping times were kept for analysis. For each patient, the total ischemia duration between clamping and declamping time was calculated. A linear regression model was used to compare the calculated and the reported ischemia duration. Results within the 0.95 confidence interval were considered as cross-validated of the reported values for ischemia duration with clamping and declamping times. A correlation of 0.99 between the reported and calculated values was obtained.

### 2.3. Data Sources

Donor characteristics and post-transplant outcomes were retrieved from the Cristal registry. Recipient comorbidities and pretransplant dialysis modalities were extracted from The French Renal Epidemiology and Information Network (REIN) registry [12]. Since those data are recorded at each annual follow-up for patients with renal replacement therapies (RRT) due to end-stage renal disease (ESRD), the last follow-up before transplantation was selected.

### 2.4. Statistical Analysis

Missing values were imputed by a two-stage method [13]: First, the follow-up data by the last observation carry forward method were imputed. Then, multiple imputations on the selected patient follow-up variables were applied.

Categorical and continuous variables were compared using Chi-square and Student *t*-tests, respectively.

Patients were grouped according to clamping and declamping times: First, using two-hour intervals, then comparing the two periods of the day with the highest versus the lowest hazards as identified by the 2 h interval analysis, i.e., night (6 p.m.–10 a.m.) versus day-time (10 a.m.–6 p.m.) periods. The survival probabilities of these groups were calculated using the Kaplan Meier method and compared with a log rank test. For the 2 h interval model, the probability of graft failure was depicted using a sinusoidal regression model [14].

A Cox regression model was fitted to compute covariate-adjusted for graft loss and patient death only on complete cases data. Then, a backward selection of variables was performed to identify independent factors significantly associated with one-year and three-years patient and graft survival, and to provide survival curves adjusted on confounding factors, stratified on declamping time and ischemia duration.

The paired donor kidney analysis, for recipients presenting with a declamping time discrepancy, used a matched case cox regression model fitted to compute covariate-adjusted for graft and patient survival.

For all statistical tests, a *p*-value < 0.05 was considered significant.

## 3. Results

**Figure 1:**
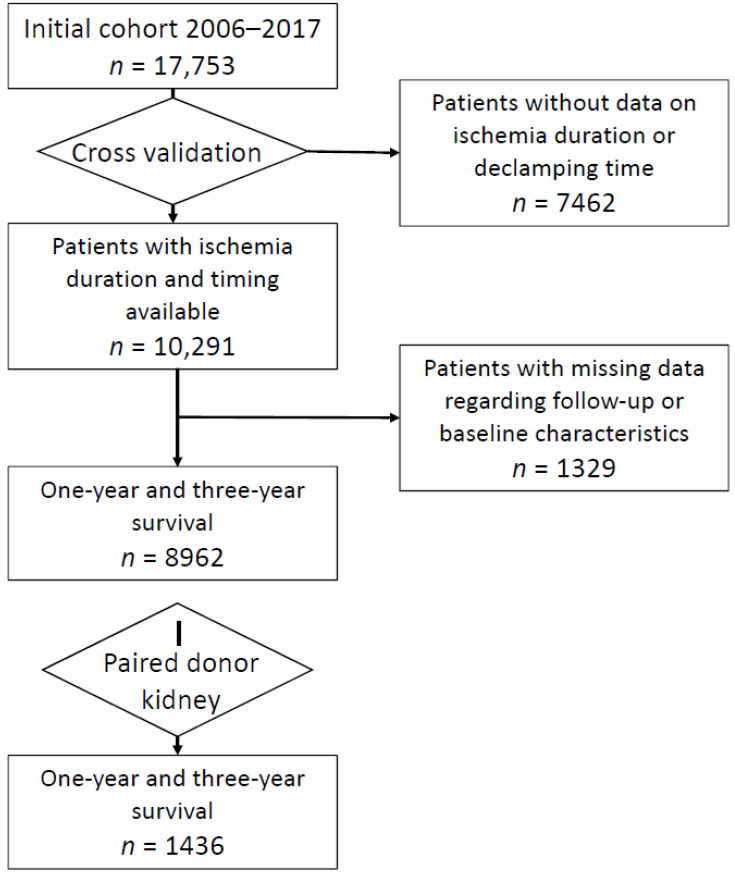
Flow chart of the study population.

Among the 17,753 renal transplanted patients, 10,291 recipients with reliable cross-validated ischemia duration, clamping and declamping times were kept for analysis (Figure 1). Baseline characteristics of this cohort are shown in Table 1. Recipients were 54 ± 14 year-old with 33% of female and 20% of diabetic patients. Most recipients (90%) displayed no or only one vascular comorbidity at the time of transplantation, with a mean duration of dialysis prior to transplantation of 3.2 ± 2.6 years.

**Figure 2:**
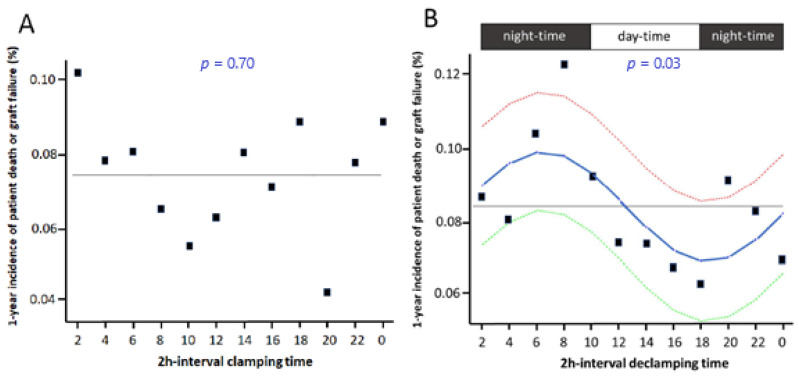
Non-adjusted 1-year survival according to clamping (**A**) and declamping (**B**) time-of-the-day. Patients were grouped by 2-h intervals according to clamping (**A**) or declamping (**B**) time-of-the-day. Square symbols: Incidence, *p*-value, and solid line by cosine function with period of 24 h by sinusoidal regression model, dashed lines: 95% confidence interval.

**Figure 3:**
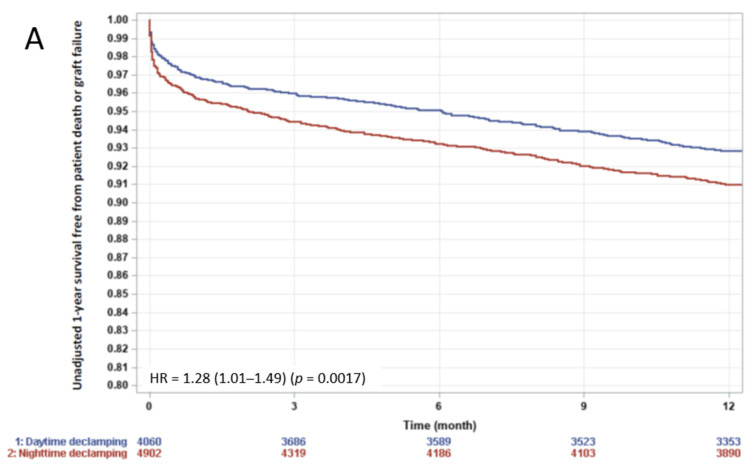

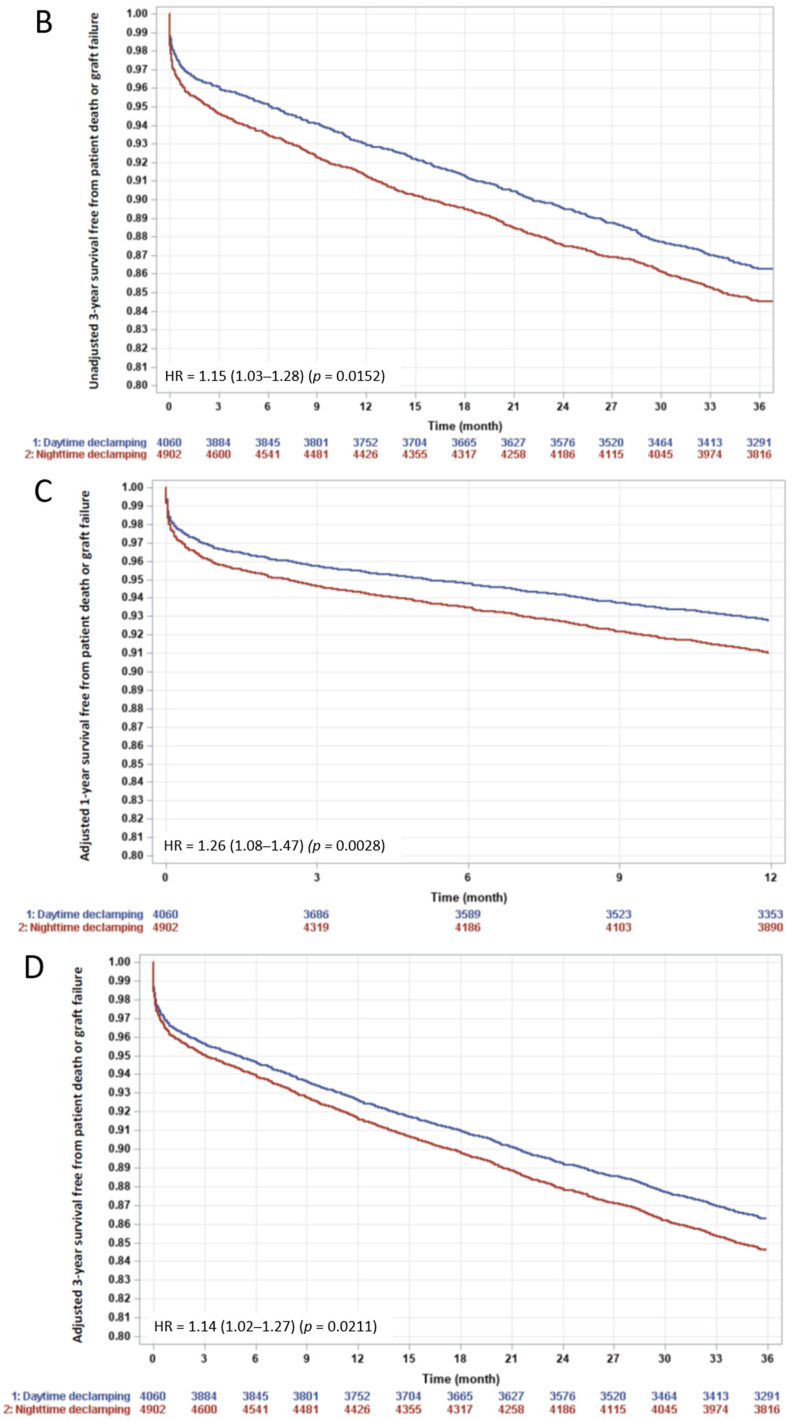
One- and 3-year survival free from patient death or graft failure after night- versus day-time declamping. Non-adjusted (**A**,**B**) and adjusted (**C**,**D**) survival curves. Day-time declamping in blue and night-time declamping in red. *p*-value by log-rank test.

Clamping time-of-the-day did not influence survival as assessed using a 2 h interval model (*p* = 0.70 by sinusoidal regression model) (Figure 2A) or comparing night- vs. day-time (*p* = 0.63). Conversely, declamping time-of-the-day impacted on the risk of graft failure as assessed using the 2-h interval model (*p* = 0.03 by sinusoidal regression model) (Figure 2B). The two periods of the day with the highest versus the lowest hazards were identified by the 2-h interval analysis, i.e., night- (6 p.m.–10 a.m.) versus day-time (10 a.m.–6 p.m.) periods. Night-time declamping (vs. day-time) was associated with a significantly increased patient death or graft failure rate at 1-year (Figure 3A, HR = 1.28 (1.01–1.49), *p* = 0.0017) and 3-year (Figure 3B, HR = 1.15 (1.03–1.28), *p* = 0.0152). Causes of death and graft loss, especially acute rejection and chronic allograft nephropathy, were similar between the two groups (Supplementary Table S1), arguing against a different contribution of the allo-immune response when declamping time occurred in the day- versus night-time. No difference was observed regarding primary graft nonfunction.

Comparison of the day-time and night-time groups showed slight, but significant, differences in CPRA, number of cardio-vascular comorbidities, dialysis duration, number of HLA DQB and HLA DR mismatches (*p* < 0.05) (Table 1). Interestingly, ischemia duration was longer in the day-time (17.5 ± 6.6 h) than in the night-time declamping group (16.5 ± 5.3 h, *p* < 0.001). Donor characteristics were similar between the two groups with a Kidney Donor Risk Index at 1.85 ± 0.71 vs. 1.84 ± 0.71 (*p* = 0.617) in the night- vs. day-time declamping group, respectively.

A multivariate Cox model was built to identify independent risk factors for post-transplant survival. Age, BMI, pre-transplant dialysis duration, Kidney Donor Risk Index, ischemia duration and declamping time-of-the-day were significantly associated with 1- and 3-year post-transplant patient or graft survival (Table 2). After adjustment for these co-variables, night-time (vs. day-time) declamping remained associated with a worse 1-year (HR= 1.26 (1.08–1.47), *p* = 0.0028) and 3-year (HR= 1.14 (1.02–1.27), *p* = 0.021) outcome (Figure 3C,D).

**Figure 4:**
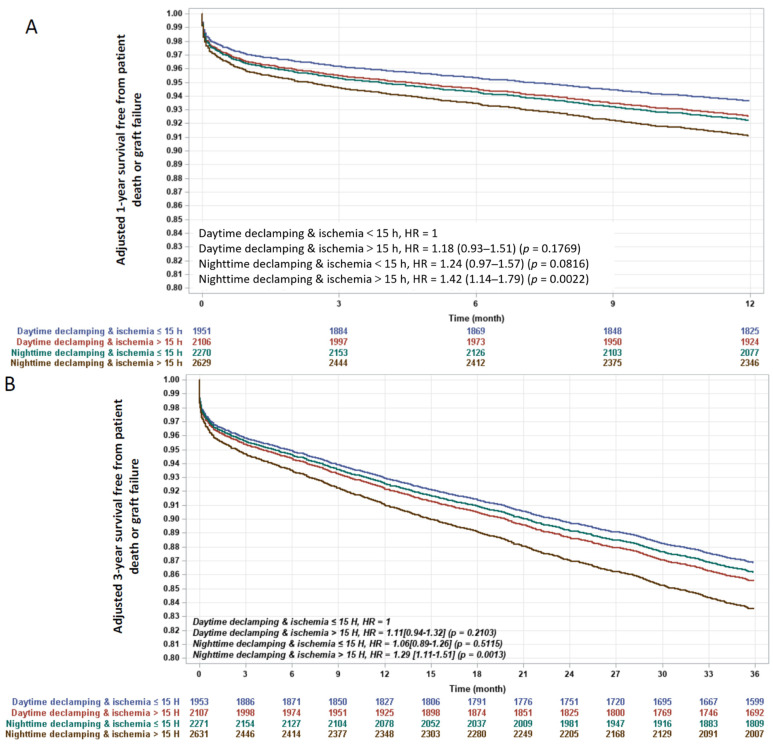
Adjusted 1- (**A**) and 3-year (**B**) survival curves according to ischemia duration and declamping time-of-the-day. *p*-value and Hazard Ratio [95%CI] by multivariate Cox-model. Day-time declamping and ischemia <15 h in blue (ischemia duration = 12.3 ± 2.2 h); day-time declamping and ischemia > 15 h in red (ischemia duration = 22.2 ± 5.6 h); night-time declamping and ischemia <15 h in green (ischemia duration = 12.2 ± 2.3 h); night-time declamping and ischemia >15 h in black (ischemia duration = 20.2 ± 4.0 h).

Interestingly, when testing the interaction between declamping time-of-the-day and ischemia duration after adjustment for the other predictors, the combination of day-time declamping with short ischemia duration (<15 h) was associated with the best 1-year outcome (Figure 4A), while night-time declamping with prolonged ischemia duration (≥15 h) was associated with the worst outcome. Survival rates of day-time declamping with prolonged ischemia duration and night-time declamping with short ischemia duration were very similar and in between the other survival curves.

Night declamping with prolonged ischemia duration (≥15 h) remained the worst combination after three years, while ischemia duration ≥ 15 h did not impact outcomes in case of day-time declamping (Figure 4B).

After one year, patient death or graft failure event rate was 77.2[68.6–86.8] vs. 98.6[89.6–108.6] per 1000 transplantations after day- vs. night-time declamping.

No week-end effect was observed regarding cold ischemia duration (data not shown). Moreover, we tested a weekend effect by enforcing the variable “week-day vs. weekend” in the multivariate Cox model presented in Table 2. We found no significant week-end effect on 1-year outcome (HR[CI95%] = 0.9[0.93–1.3] vs. week-day, *p* = 0.27) while the time-of-the-day effect remained significant, (HR(CI95%) = 1.26 (1.08–1.46) vs. day-time declamping, *p* = 0.0037).

### Sub-Group Analysis in the Paired Patients Transplanted with Kidneys from the Same Donor

A paired kidney analysis was performed for recipients who presented declamping time-of-the-day differences (*n* = 1436, Supplementary Table S2 for patient characteristics). Night-time declamping was associated with worse 1-year post-transplant patient and graft survival before (HR = 1.44(0.99–2.1); *p* = 0.058) and borderline significant after adjustment for other predictors (HR= 1.49 (0.99–2.22); *p*= 0.053), findings which were consistent with the analysis of the entire cohort.

## 4. Discussion

Exploring a large cohort of patients who underwent kidney transplantation, we show that, whereas time-of-the-day of clamping does not impact patient and graft survival, day-time (vs. night-time) declamping was associated with better post-transplant survival rate, irrespective of ischemia duration and other confounding factors. Interestingly, the interaction between declamping time-of-the-day and ischemia duration suggests that the detrimental effect of prolonged ischemia duration was partly compensated by day-time declamping.

Although the results from the paired kidney analysis, which considerably limits the confounding factors related to the donor, were consistent with the entire cohort, the survival rate did not reach statistical significance, which could be related to a loss of statistical power in this subgroup analysis.

Several mechanisms can be hypothesized to explain the present observation. First, a human factor can be at play: Less efficiency of the surgeon and/or medical care in the night period might induce prolonged ischemia duration and higher rates of technical failure. The few studies that previously investigated the impact of off-duty kidney transplantation did not show consistent data supporting such a fatigue effect [2,3,4,5,6,7,8]. Although Flechner et al. showed that transplantations performed between 8 pm and 8 am result in a higher risk for 5-year graft failure [2], Özdemir-van Brunschot et al. did not report such an off-hour impact in a larger population with an unexpected lower incidence of pure technical graft failure at night [3]. In our study, cold ischemia duration was shorter for kidneys transplanted at night. The implantation surgical team was not the team that performed the organ procurement. Moreover, the sinusoidal analysis demonstrates that the highest risk for allograft loss was associated with an 8 am declamping time, which would be the start of the operating day. This is thus less likely to be associated with a fatigued surgeon.

Second, the IR injury and repair processes after renal transplantation may display circadian rhythmicity. Many biological processes are paced at a 24-h circadian rhythm, which is synchronized by external stimuli (Zeitgebers) resetting endogenous molecular clocks, consisting of a series of interlocked transcriptional/translational feedback loops that generate and maintain the organism’s rhythm [15]. Renal blood flow, glomerular filtration rate and various tubular functions involved in secretion and reabsorption, and hormone production exhibit a diurnal variation [16]. Moreover, modulation of clock gene expression alters not only IR tolerance, but also subsequent adverse organ remodeling by inflammatory/fibrotic processes. Recently, Sun et al. demonstrated a diurnal variability of renal injury induced by IR in a rat model of 45-min bilateral occlusion of renal pedicles [17]. Histo-pathological changes following 24 h of reperfusion were worst in case of ischemia performed at the sleep-to-wake (vs. wake-to-sleep) transition. Circadian clock regulation of the protective nuclear erythroid 2-related factor2 (NRF2)/anti-oxidant response element (ARE) pathway was evoked as one potential mechanism. IR tolerance is the lowest at the time of sleep-to-wake transition, i.e., time of high expression of the clock gene Rev-erbα, which regulates NLRP3 expression and production of inflammatory cytokines in monocytes and macrophages [18,19], and low expression of RORα in rodent and human myocardium [20] as well as in rodent kidney [21]. In line, Cunningham et al. demonstrated that the incidence of primary graft dysfunction after lung transplantation depends on the timing of allograft implantation: Lungs reperfused between 4 and 8 am had a higher incidence of primary graft dysfunction, most probably as a result of the circadian regulation of post-transplant immune activation [22]. Thus, modulating the circadian clock with pharmacological tools, such as RORα and Rev-erbα nuclear receptor agonists/antagonists, would appear a promising approach to improve kidney graft survival and prognosis, providing a causal role for the circadian clock is demonstrated in the future.

## 5. Limitations

Several limitations are inherent in the use of registry data.

Despite a large number of variables in the Cristal registry, it is impossible to certify that all possible confounders have been considered in this retrospective study. Surgeon and medical staff experience, as well as post-operative care, were not reported in the registry. Along the same line, length of surgical time was unfortunately unavailable in the Cristal registry, which represents a potential confounding factor, and might contribute to the differences observed between the clamping and declamping correlations.

Slight differences were observed between day-time and night-time declamping groups regarding vascular comorbidities history, duration of dialysis, CPRA and subsequently HLA compatibility, ischemia duration. Despite being statistically significant, these differences were of little clinical relevance and survival curves were adjusted for these potential confounders, identified in the Cox model, to limit bias.

The study population was restricted to the patients with reliable cross-validated ischemia duration, clamping and declamping times. Although we cannot totally exclude a sample effect, we assumed that coding errors and missing data occur at random.

Information on the experience of the medical staff, specifically the surgeon performing the graft transplantations is not available in the Cristal database.

A causal relation cannot be demonstrated by our study design. At this stage, although day-time clamping appears to be associated with better results, this does not necessarily imply that a delay in transplant surgery would improve outcomes.

## 6. Conclusions

Day-time declamping is associated with better graft survival rates after kidney transplantation compared with night-time declamping, despite a longer duration of cold ischemia. Future research will aim at replicating this observation and elucidating its precise underlying mechanisms in a view to enhance graft survival and consequently decrease graft-failure-related costs.

## Figures and Tables

**Table 1:** Baseline characteristics of the studied cohort (*n* = 8962) and grouped according to declamping time-of-the-day. BMI, body mass index; CPRA, calculated panel reactive antibody; HLA, human leukocyte antigen; *p*-value for night-time vs. day-time declamping by *t*-test or Chi-square test.

		All	Day-Time Declamping	Night-Time Declamping	*p*-Value
		(*n* = 8962)	(*n* = 4060)	(*n* = 4902)	
Female gender		3318 (37.0%)	1497 (36.9%)	1821 (37.1%)	0.788
Patient age	mean (std)	54.3 (13.7)	54.2 (13.9)	54.4 (13.5)	0.508
BMI	<25	4419 (49.3%)	2045 (50.4%)	2374 (48.4%)	0.135
	25–29	2967 (33.1%)	1329 (32.7%)	1638 (33.4%)	
	≥30	1576 (17.6%)	686 (16.9%)	890 (18.2%)	
Diabetes		1716 (19.1%)	761 (18.7%)	955 (19.5%)	0.377
Vascular comorbidity	0 Vascular comorbidity	6384 (71.2%)	2956 (72.8%)	3428 (69.9%)	0.028
	1 Vascular comorbidity	1666 (18.6%)	716 (17.6%)	950 (19.4%)	
	2 Vascular comorbidities	625 (7.0%)	268 (6.6%)	357 (7.3%)	
	≥3 Vascular comorbidities	287 (3.2%)	120 (3.0%)	167 (3.4%)	
Paraplegia/Hemiplegia		77 (0.9%)	30 (0.7%)	47 (1.0%)	0.262
Blindness		197 (2.2%)	90 (2.2%)	107 (2.2%)	0.913
Malignancy		269 (3.0%)	119 (2.9%)	150 (3.1%)	0.722
Behavioral disorders		125 (1.4%)	56 (1.4%)	69 (1.4%)	0.910
Walk ability	Total incapacity	43 (0.5%)	22 (0.5%)	21 (0.4%)	0.531
	Need for help	111 (1.2%)	46 (1.1%)	65 (1.3%)	
	Independent	8808 (98.3%)	3992 (98.3%)	4816 (98.2%)	
Chronic Respiratory Insufficiency		506 (5.6%)	230 (5.7%)	276 (5.6%)	0.944
Albumin	mean (std)	38.25 (4.90)	38.33 (5.00)	38.19 (4.82)	0.186
CPRA	mean (std)	15.98 (29.27)	13.22 (26.78)	18.27 (31.00)	<0.001
Dialysis Modality	Assisted dialysis	4651 (51.9%)	2082 (51.3%)	2569 (52.4%)	0.288
Duration of dialysis (y)	mean (std)	3.16 (2.64)	3.01 (2.46)	3.28 (2.78)	<0.001
Pre-emptive Transplant		182 (2.0%)	86 (2.1%)	96 2.0%)	0.593
Total ischemia duration (h)	mean (std)	16.94 (5.94)	17.47 (6.57)	16.50 (5.32)	<0.001
Number of HLA A mismatches	0	1189 (13.3%)	525 (12.9%)	664 (13.5%)	0.580
	1	4609 (51.4%)	2109 (51.9%)	2500 (51.0%)	
	2	3164 (35.3%)	1426 (35.1%)	1738 (35.5%)	
Number of HLA B mismatches	0	633 (7.1%)	266 (6.6%)	367 (7.5%)	0.182
	1	3684 (41.1%)	1663 (41.0%)	2021 (41.2%)	
	2	4645 (51.8%)	2131 (52.5%)	2514 (51.3%)	
Number of HLA DQ B mismatches	0	4588 (51.2%)	1995 (49.1%)	2593 (52.9%)	0.001
	1	3892 (43.4%)	1848 (45.5%)	2044 (41.7%)	
	2	482 (5.4%)	217 (5.3%)	265 (5.4%)	
Number of HLA DR mismatches	0	2760 (30.8%)	1178 (29.0%)	1582 (32.3%)	<0.001
	1	4841 (54.0%)	2218 (54.6%)	2623 (53.5%)	
	2	1361 (15.2%)	664 (16.4%)	697 (14.2%)	

**Table 2:** Factors associated with 1 and 3-year survival (i.e., patient or non-death censored graft survival) according to the multivariate Cox Model.

	One-Year Survival				Three-Year Survival		
Factors	Value	*n* (%)	HR (CI95%)	*p*-Value	*n* (%)	HR (CI95%)	*p*-Value
Patient’s age (y)	Continuous	8962 (100%)	1.01 (1–1.02)	0.0045	8962 (100%)	1.01 (1–1.01)	0.0218
Albumin (g/L)	Continuous	8962 (100%)	0.98 (0.97–1)	0.0383	8962 (100%)	0.98 (0.97–0.99)	0.0037
Patient BMI	<25	4419 (49.31%)	1		4419 (49.31%)	1	
	25–29	2967 (33.11%)	1.14 (0.96–1.36)	0.1343	2967 (33.11%)	1.05 (0.92–1.19)	0.4506
	≥30	1576 (17.59%)	1.52 (1.25–1.85)	<0.0001	1576 (17.59%)	1.46 (1.27–1.69)	<0.0001
Kidney Donor Risk Index	Continuous	8962 (100%)	1.57 (1.37–1.81)	<0.0001	8962 (100%)	1.74 (1.57–1.93)	<0.0001
Number of Cardio-Vascular Comorbidities	0	6384 (71.23%)	1		6384 (71.23%)	1	
	1	1666 (18.59%)	1.31 (1.09–1.58)	0.0046	1666 (18.59%)	1.27 (1.11–1.46)	0.0006
	2	625 (6.97%)	1.95 (1.55–2.45)	<0.0001	625 (6.97%)	1.88 (1.58–2.23)	<0.0001
	≥3	287 (3.2%)	1.74 (1.27–2.4)	0.0006	287 (3.2%)	1.65 (1.3–2.1)	<0.0001
Dialysis duration	Continuous	8962 (100%)	1.05 (1.02–1.07)	0.0002	8962 (100%)	1.04 (1.02–1.06)	<0.0001
Declamping time	Day-time declamping	4060 (45.3%)	1		4060 (45.3%)	1	
	Night-time declamping	4902 (54.7%)	1.26 (1.08–1.47)	0.0028	4902 (54.7%)	1.14 (1.02–1.27)	0.0211
Ischemia duration (h)	Continuous	8962 (100%)	1.02 (1.01–1.03)	0.0012	8962 (100%)	1.02 (1.01–1.02)	0.0014
Patient gender	Female				3318 (37.02%)	1	
	Male				5644 (62.98%)	1.17 (1.04–1.31)	0.0109
Dialysis modality	Non-assisted dialysis				4311 (48.1%)	1	0.0165
	Assisted dialysis				4651 (51.9%)	1.15 (1.03–1.28)	

## Data Availability

Data availability is under the responsibility of C.J. (Agence de la Biomedecine, France).

## Supplementary Materials

The following are available online at https://www.mdpi.com/article/10.3390/jcm10112322/s1. Table S1: Cause of graft failure and death according to time-of-the day of declamping. No statistical difference was observed between the 2 groups. Table S2: Baseline characteristics of the sub-population of renal transplanted patients paired on the same donor (*n* = 1436) and grouped according to declamping time-of-the-day. BMI, body mass index; CPRA, calculated panel reactive antibody. p-value for night-time vs day-time declamping by *t*-test or Chi-square test.
