# Supplementary material for: Day-Time Declamping Is Associated with Better Outcomes in Kidney Transplantation: The Circarein Study
Source: J Clin Med. 2021 May 26;10(11):2322. doi: 10.3390/jcm10112322 (PMC8198093; doi:10.3390/jcm10112322)
Supplement: Supplementary file 1 [file jcm-10-02322-s001.zip › jcm-1211834-supplementary.pdf]

Supplementary Table S1. Cause of graft failure and death according to time-of-the day of declamping. No statistical difference was observed between the 2 groups.

**Cause of graft failure**	**Day-time declamping**	**(%)**	**Night-time declamping**	**(%)**
Chronic allograft nephropathy	106	59,2	160	59,9
Primary non function	17	9,5	27	10,1
Acute rejection	17	9,5	20	7,5
Haemorrage related to surgical procedure	3	1,7	6	2,2
Multiorgan failure	5	2,8	6	2,2
Graft urologic complication	4	2,2	6	2,2
Infection	7	3,9	15	5,6
Initial diease recurrence	4	2,2	10	3,7
Treatment withdrawal	1	0,6	3	1,1
Others	15	8,4	13	4,9
Missing	0	0,0	1	0,4
**Cause of death**	**Day-time declamping (n=99)**	**(%)**	**Night-time declamping (n=149)**	**(%)**
Cardiovascular death	36	36,4	58	38,9
Infection	16	16,2	31	20,8
Graft related death	7	7,1	11	7,4
Malignancy	7	7,1	6	4,0
Digestive cause	3	3,0	2	1,3
Psychiatric complication	3	3,0	2	1,3
Other non-cardiovascular death	14	14,1	16	10,7
Unknown cause	13	13,1	22	14,8
Missing	0	0,0	1	0,7

Supplementary Table S2. Baseline characteristics of the sub-population of renal transplanted patients paired on the same donor (n=1436) and grouped according to declamping time-of-the-day. BMI, body mass index; CPRA, calculated panel reactive antibody. p-value for night-time vs day-time declamping by t-test or Chi-square test.

		**Total** (*n* = 1436)	**Day-time declamping** (*n* = 718)	**Night-time declamping** (*n* = 718)	***p*-Value**
**Female gender**		**534 (37.2%)**	248 (34.5%)	286 (39.8%)	0.038
Patient age	mean (std)	56.05 (13.28)	56.33 (13.48)	55.78 (13.07)	0.439
	< 25	664 (46.2%)	341 (47.5%)	323 (45.0%)	0.631
BMI	25-29	496 (34.5%)	243 (33.8%)	253 (35.2%)	
	≥ 30	276 (19.2%)	134 (18.7%)	142 (19.8%)	
Diabetes		313 (21.8%)	142 (19.8%)	171 (23.8%)	0.064
Vascular comorbidity	0 Vascular comorbidity	987 (68.7%)	501 (69.8%)	486 (67.7%)	0.477
	1 Vascular comorbidity	261 (18.2%)	128 (17.8%)	133 (18.5%)	
	2 Vascular comorbidities	129 (9.0%)	57 (7.9%)	72 (10.0%)	
	≥ 3 Vascular comorbidities	59 (4.1%)	32 (4.5%)	27 (3.8%)	
Paraplegia / Hemiplegia		13 (0.9%)	6 (0.8%)	7 (1.0%)	0.781
Blindness		21 (1.5%)	13 (1.8%)	8 (1.1%)	0.272
Evolutionary cancer		49 (3.4%)	25 (3.5%)	24 (3.3%)	0.884
Behavioral disorders		20 (1.4%)	12 (1.7%)	8 (1.1%)	0.368
	Total incapacity	6 (0.4%)	4 (0.6%)	2 (0.3%)	0.477
Walk ability	Need for help	20 (1.4%)	12 (1.7%)	8 (1.1%)	
	Independent	1410 (98.2%)	702 (97.8%)	708 (98.6%)	
Chronic Respiratory Insufficiency		90 (6.3%)	41 (5.7%)	49 (6.8%)	0.384
Albumen	mean (std)	38.31 (4.78)	38.46 (4.77)	38.17 (4.79)	0.253
CPRA	mean (std)	16.26 (29.70)	12.27 (26.06)	20.26 (32.48)	<0.001
Dialysis Modality	Assisted dialysis	733 (51.0%)	366 (51.0%)	367 (51.1%)	0.958
Duration of dialysis (y)	mean (std)	3.13 (2.69)	2.97 (2.45)	3.28 (2.91)	0.030
Pre-emptive Transplant		29 (2.0%)	12 (1.7%)	17 (2.4%)	0.348
Total ischemia duration (h)	mean (std)	17.47 (6.31)	17.20 (7.04)	17.74 (5.48)	0.108
	0	181 (12.6%)	84 (11.7%)	97 (13.5%)	0.473
Number of HLA A mismatches	1	720 (50.1%)	358 (49.9%)	362 (50.4%)	
	2	535 (37.3%)	276 (38.4%)	259 (36.1%)	
	0	97 (6.8%)	34 (4.7%)	63 (8.8%)	0.003
Number of HLA B mismatches	1	572 (39.8%)	278 (38.7%)	294 (40.9%)	
	2	767 (53.4%)	406 (56.5%)	361 (50.3%)	
	0	744 (51.8%)	356 (49.6%)	388 (54.0%)	0.227
Number of HLA DQB mismatches	1	620 (43.2%)	323 (45.0%)	297 (41.4%)	
	2	72 (5.0%)	39 (5.4%)	33 (4.6%)	
	0	463 (32.2%)	209 (29.1%)	254 (35.4%)	0.001
Number of HLA DR mismatches	1	771 (53.7%)	387 (53.9%)	384 (53.5%)	
	2	202 (14.1%)	122 (17.0%)	80 (11.1%)	
